# The Effect of Bariatric Surgery on Circulating Levels of Oxidized Low-Density Lipoproteins Is Apparently Independent of Changes in Body Mass Index: A Systematic Review and Meta-Analysis

**DOI:** 10.1155/2021/4136071

**Published:** 2021-12-06

**Authors:** Tannaz Jamialahmadi, Željko Reiner, Mona Alidadi, Matthew Kroh, Vladimiro Cardenia, Suowen Xu, Khalid Al-Rasadi, Raul D. Santos, Amirhossein Sahebkar

**Affiliations:** ^1^Department of Nutrition, Faculty of Medicine, Mashhad University of Medical Sciences, Mashhad, Iran; ^2^Department of Internal Medicine, University Hospital Centre Zagreb, School of Medicine, University of Zagreb, Zagreb, Croatia; ^3^Digestive Disease and Surgery Institute, Cleveland Clinic Lerner College of Medicine, Cleveland, Ohio, USA; ^4^Department of Agricultural, Forest and Food Sciences (DISAFA), University of Turin, Grugliasco (TO) 10095, Italy; ^5^Division of Life Sciences and Medicine, University of Science and Technology of China, Hefei, China; ^6^Medical Research Centre, Sultan Qaboos University, Muscat, Oman; ^7^Lipid Clinic Heart Institute (Incor), University of São Paulo, Medical School Hospital, São Paulo, Brazil; ^8^Applied Biomedical Research Center, Mashhad University of Medical Sciences, Mashhad, Iran; ^9^Biotechnology Research Center, Pharmaceutical Technology Institute, Mashhad University of Medical Sciences, Mashhad, Iran; ^10^Department of Medical Biotechnology, Faculty of Medicine, Mashhad University of Medical Sciences, Mashhad, Iran; ^11^Department of Biotechnology, School of Pharmacy, Mashhad University of Medical Sciences, Mashhad, Iran

## Abstract

**Background:**

Obesity is related to dyslipidemia and increased circulating oxidated LDL (ox-LDL) concentrations that may predispose to atherosclerosis. Bariatric surgery may lower the risk of cardiovascular mortality. Elevated plasma ox-LDL has been associated with atherogenesis and atherosclerotic cardiovascular disease (ASCVD) events. The aim of this meta-analysis was to investigate the impact of bariatric surgery on proatherogenic circulating ox-LDL levels in patients with severe obesity.

**Methods:**

Four databases were systematically searched from inception to May 1, 2021. Also, to clarify the heterogeneity of studies with regard to treatment duration, research design, and the demographic features, a random-effects model and the generic inverse variance weighting approach were utilized. To determine the association with the estimated effect size, a random-effect meta-regression approach was performed. Finally, a meta-regression analysis was conducted to explore the influence of, respectively, baseline and changes in body mass index (BMI), baseline ox-LDL, and postsurgery follow-up period with the estimated effect size of surgery on ox-LDL levels.

**Results:**

Meta-analysis of 11 studies including 470 subjects showed a significant decline in circulating ox-LDL following bariatric surgery (SMD: -0.971, 95% CI: -1.317, -0.626, *p* < 0.001, *I*^2^: 89.43%). The results of meta-regression did not show any significant association between the changes in ox-LDL after bariatric surgery and baseline BMI, duration of follow-up or baseline ox-LDL values. However, there was a significant association between ox-LDL alteration and percentage of BMI change.

**Conclusion:**

Bariatric surgery in patients who had severe obesity causes a decrease of circulating ox-LDL that was apparently dependent in BMI changes.

## 1. Introduction

Obesity is a major risk factor for impaired glucose tolerance, insulin resistance, and type 2 diabetes mellitus, particularly atherosclerotic cardiovascular disease (ASCVD) [1]. Obesity is associated with atherogenic dyslipidemia, low-grade inflammation, and an overall prothrombotic state [2–4]. Dyslipidemia, and especially elevated plasma low-density lipoprotein (LDL) cholesterol, is a pivotal risk factor for atherosclerosis [5]. It is well established that atherogenesis begins with endothelial dysfunction or damage. When LDL particles rich in cholesterol are present in plasma in larger quantities, they permeate through the altered endothelium into the subendothelial space entering the intima. Once this occurs, the LDL particles are oxidized by reactive oxygen species (ROS) and modified into oxidated LDL (ox-LDL) [6, 7]. Ox-LDL particles are strong ligands for macrophage scavenger receptors (CD36, SR-AI/II, and SR-BI) allowing them to enter macrophages and transform them into foam cells [8]. Foam cells, when piled up, appear macroscopically as fatty streaks which are an important step towards fibro-lipid atherosclerotic plaques build-up. Therefore, it is not surprising that increased circulating ox-LDL levels are linked to clinical ASCVD events [9].

Bariatric surgery is a durable and effective therapeutic approach in severely obese individuals. Most endocrinology societies recommend surgical therapy for individuals with BMI ≥ 40 kg/m^2^ or for those with a BMI ranging from 35 to 39.9 kg/m^2^ and comorbidities who may benefit from weight reduction, as well as for severe obese individuals with a BMI 30.0–34.9 kg/m^2^ and poorly controlled type 2 diabetes mellitus. The most common types of bariatric surgery are sleeve gastrectomy (SG), Roux-en-Y gastric bypass (RYGP), one anastomosis gastric bypass/mini gastric bypass (OAGB/MGB), laparoscopic adjustable gastric band (LAGB), and biliopancreatic diversion/duodenal switch (BPD/DS) [10]. Weight loss following bariatric surgery can lower ASCVD risk as well as ensuing mortality in severely obese individuals [11–15].

Since severe obesity is associated with dyslipidemia and increased LDL oxidation that may predispose to atherosclerosis and its ominous consequences, it would be important to verify if bariatric surgery would reduce oxidative stress.

Therefore, the aim of this systematic review and meta-analysis was to establish the effect of bariatric surgery on levels of circulating ox-LDL.

## 2. Methods

### 2.1. Search Strategy

This systematic review and meta-analysis were done based on the 2009 preferred reporting items for systematic reviews and meta-analysis (PRISMA) guidelines [16]. PubMed, Embase, Scopus, and Web of Science were searched from inception to May 1, 2021, using keywords in abstracts and titles (also in combination with MESH terms) as follows: (“bariatric surgery” OR gastroplast∗ OR “gastric bypass” OR “Roux-en-Y” OR “gastric band” OR “biliopancreatic diversion” OR gastrectom∗ OR “duodenal switch” OR “gastrointestinal diversion” OR gastroenterostom∗ OR “jejunoileal bypass” OR “obesity surgery” OR “weight loss surgery” OR “weight-loss surgery” OR “bariatric procedure” OR “sleeve surgery” OR “metabolic surgery”) AND (“oxidized low density lipoprotein” OR “oxidized LDL” OR “OxLDL” OR “ox-LDL” OR “oxidized Low-Density Lipoprotein” OR “minimally modified oxidized-LDL” OR “MM-LDL” OR “MMLDL” OR “malondialdehyde-low density lipoprotein” OR “malondialdehyde low density lipoprotein” OR “MDA-LDL” OR “MDALDL”).

### 2.2. Study Selection

For inclusion, only original peer-reviewed studies written in English language were considered. All forms of bariatric surgery procedures were taken into account. Articles must have reported circulating ox-LDL before and after surgery. The exclusion criteria were only abstracts, letters, case reports, comments, meta-analyses, duplicate studies, animal studies, reviews, non-English papers, studies with no surgical intervention, and studies without outcomes.

### 2.3. Data Extraction

Following the removal of duplicate research, two independent authors reviewed the abstracts and titles of the remaining papers for inclusion. The whole texts of the applicable studies were collected. When two papers with the same research purpose were published by the same organization and/or authors, the study published more recently with a larger sample size was included. Any differences were discussed by authors. The following data was gathered from studies which were eligible for inclusion: (1) the name of the first author, (2) the year of publication, (3) the type of surgery, (4) the study design, (5) the characteristics of the patients, (6) oxidized LDL levels, and (7) the period of follow-up.

### 2.4. Quality Assessment

The Newcastle-Ottawa scale (NOS) was performed to evaluate the study quality in this meta-analysis [17]. Three features of each eligible study are taken into account for this scale: (1) the selection of the studied patients (4 items), (2) the comparability of the studied populations (1 item), and (3) the ascertainment of the exposure (3 items) in case-control studies or outcome of interest in cohort studies.

### 2.5. Quantitative Data Synthesis

Meta-analysis was performed using Comprehensive Meta-Analysis (CMA) V2 software (Biostat, NJ) [18]. Information regarding sample size, means, and standard deviations from each group were extracted to calculate the standardized mean differences (SMDs). SMD was applied since several different assays were utilized to determine plasma ox-LDL levels. Random-effects meta-analysis was used to get overall estimate of effect size. Postoperative mean and SD were used to calculate the final effect size. To clarify the heterogeneity of studies regarding treatment duration, design of study, and the characteristics of the studied populations, a random-effects model (owing to interstudy heterogeneity) and the generic inverse variance weighting method were utilized [16]. Clinical heterogeneity was judged by study locations and recruited populations, methods applied for ox-LDL assay, baseline ox-LDL values, and differences in biochemical parameters among studied populations. Statistical heterogeneity was appraised by *I*^2^ index and Cochrane's *Q* test. The mean and standard deviation were calculated using the method described by Hozo et al. if the outcome measures were reported as median and range (or 95 percent confidence interval (CI)) [19]. When only standard error of the mean (SEM) was supplied, SD was calculated using the following formula: SD = SEM sqrt (*n*), where “*n*” denotes the number of participants. A sensitivity analysis using the leave-one-out approach was carried out to explore the impact of each study on the overall effect size (i.e., deleting one study each time and repeating the analysis) [20, 21].

### 2.6. Meta-Regression

A meta-regression analysis was carried out to investigate the impact of, respectively, baseline and changes in BMI, baseline ox-LDL and duration of postsurgery follow-up with the estimated effect size of surgery on ox-LDL concentrations.

### 2.7. Subgroup Analysis

A subgroup analysis was conducted to investigate the impact of follow-up duration (≥12 months and <12 months) with the estimated effect size of surgery on ox-LDL concentrations.

### 2.8. GRADE Scoring

We used the grade of recommendations, assessment, development, and evaluation (GRADE) approach to assess the strength of evidence for each outcome [22]. To summarize the findings for each outcome, the GRADEpro GDT software was used. We assigned four points to each outcome and then evaluated factors that reduced the quality of the evidence. For each outcome, points were reduced based on the presence of the following; the overall RoB for each study, inconsistency (significant heterogeneity), indirectness (significant differences in the population, comparisons, and outcomes), and imprecision (the size of the cohort, width, and significance of the confidence intervals (CIs)). As a result, we classified the evidence into four groups depending on the aggregate GRADE ratings for each intervention: high-grade evidence (at least 4 points), moderate grade evidence (3 points), low-grade evidence (2 points), and very low-grade evidence (1 point).

### 2.9. Publication Bias

In the meta-analysis, the funnel plot was used to realize the presence of publication bias. Hence, Begg's rank correlation and Egger's weighted regression tests were often performed to help publication bias detection. When asymmetry in the funnel plot was found, potentially missing studies were inserted using the “trim and fill” method. In the event of a significant result, the “fail-safe N” approach was used to compute the number of potentially missing studies required to make the *p* value nonsignificant. This is another sign of publishing bias [23].

## 3. Results

**Figure 1:**
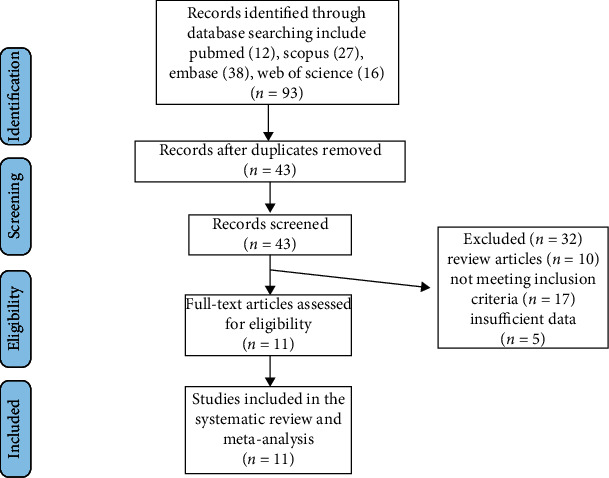
Flow chart of included studies.

A thorough database search identified 93 published papers, 43 of which were directly connected to the issue of this study. After careful consideration, 32 studies were excluded:10 studies were reviews, 17 studies did not meet the inclusion criteria, and 5 studies did not disclose enough data. As a result, 11 studies which evaluated the levels of ox-LDL after bariatric surgery were included (Table 1). The study selection procedure was indicated in Figure 1.

### 3.1. Quality Assessment of the Included Studies

All selected studies showed insufficient information for case definition, and most of them had lack of information for representativeness of the cases. Because most of the studies did not include a control group, they were not evaluated for selection of controls, definition of controls, comparability, the same method of ascertainment, and nonresponse rate. However, all studies which included met the ascertainment of exposure criteria.  Table 2 shows the details of quality assessment.

### 3.2. Assay Methods

In most of the included studies, serum ox-LDL was assessed using enzyme-linked immunosorbent assay (ELISA) method. Nine studies used Mercodia ox-LDL kit (Mercodia, Uppsala, Sweden) [24–28, 30, 31, 33, 34], one study used immunodiagnostic system (Boldon, UK) [32], and one study used OxiSelect MDA-LDL-Quantitation kit (Cell Biolabs Inc., San Diego, USA) [29].

### 3.3. Effect of Bariatric Surgery on Circulating Concentrations of Oxidized LDL

**Figure 2:**
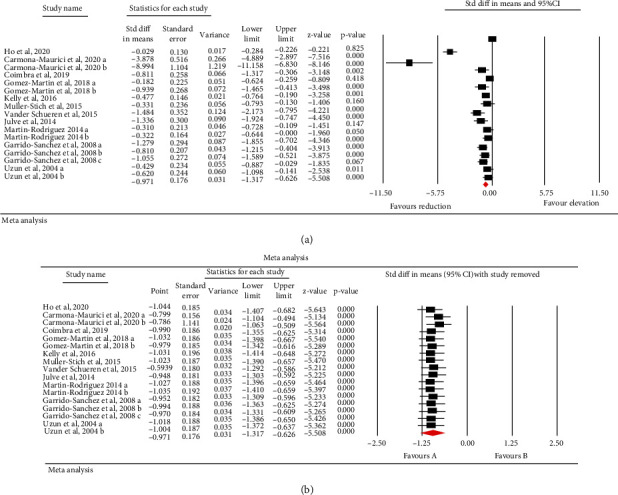
(a) Forest plot which displays weighted mean difference and 95% confidence intervals for the influence of bariatric surgery on ox-LDL. (b) Leave-one-out sensitivity analyses for the influence of bariatric surgery on ox-LDL.

Meta-analysis of 11 publications including 470 subjects demonstrated a significant reduction of circulating ox-LDL following bariatric surgery (SMD: -0.971, 95% CI: -1.317, -0.626, *p* < 0.001, *I*^2^: 89.43%) (Figure 2(a)). In the leave-one-out sensitivity analysis, the reduction in circulating ox-LDL was robust (Figure 2(b)) (low-grade evidence, Table 3).

### 3.4. Subgroup Analysis

**Figure 3:**
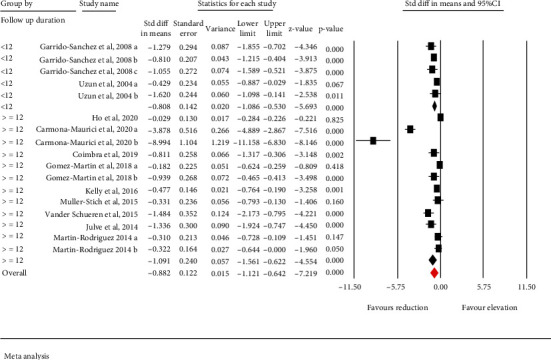
Subgroup analysis to assess the influence follow up duration in ox-LDL alteration.

A subgroup analysis was also performed based on follow-up duration (≥12 months and <12 months). Subgroup analyses demonstrated significant reduction of circulating ox-LDL following bariatric surgery in both follow-up periods (≥12 months *p* < 0.001 and <12 months *p* < 0.001). However, this analysis did not show significant associations between follow-up duration and change in ox-LDL levels (*p* = 0.309) (Figure 3).

### 3.5. Meta-Regression

**Figure 4:**
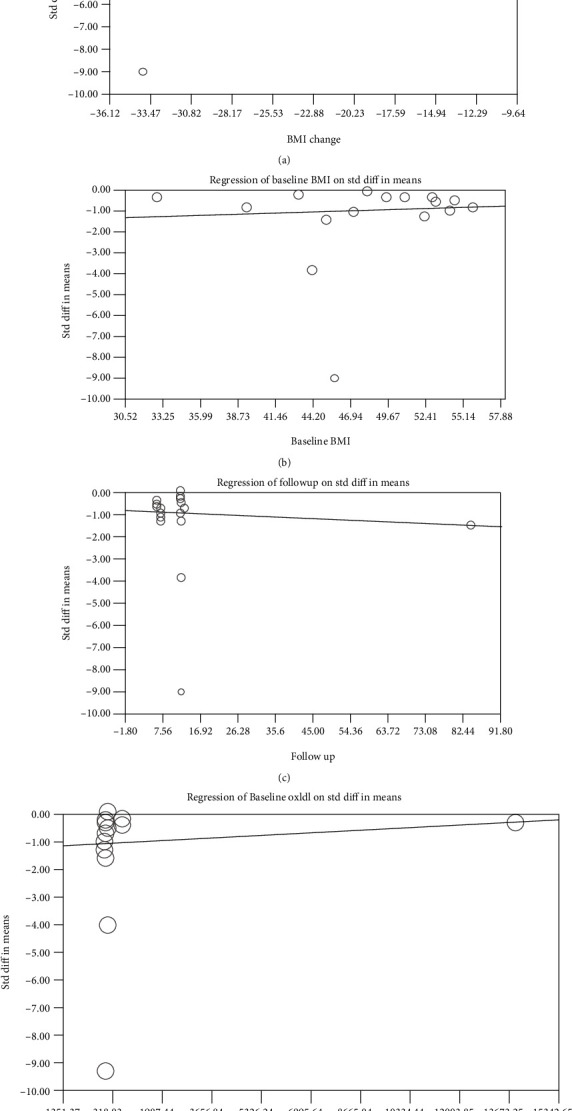
Random-effects meta-regression to assess the influence of % BMI change (a), baseline BMI (b), follow-up duration (c), and baseline oxLDL levels (d) on the estimated effect size.

Random-effects meta-regression was used to assess the effect of potential confounders on the ox-LDL reducing effect of bariatric surgery. The results did not designate any significant association between the changes in ox-LDL and baseline BMI (slope: 0.018; 95% CI: -0.041, 0.078; *p* = 0.549), follow-up duration (slope: -0.007; 95% CI: -0.028, 0.012; *p* = 0.444) or baseline ox-LDL (slope: 0.00005; 95% CI: -0.00005, 0.00016; *p* = 0.324). However, there was a significant association between changes in ox-LDL and percentage of BMI change (slope: 0.069; 95% CI: 0.003, 0.135; *p* = 0.039) (Figures 4(a)–4(d)).

### 3.6. Publication Bias

**Figure 5:**
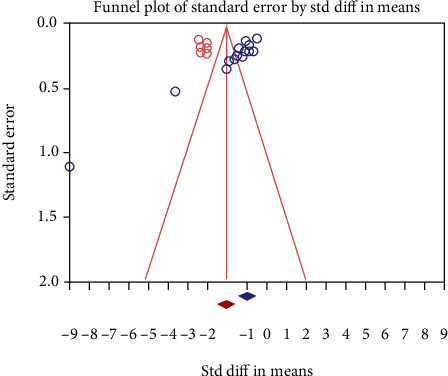
Funnel plot which displays publication bias in the studies reporting the influence of bariatric surgery on oxidized LDL.

Egger's linear regression test (intercept = −7.534, standard error = 0.93; 95%CI = −9.535, -5.533, *t* = 8.026, df = 15, two-tailed *p* < 0.001) and Begg's rank correlation test (Kendall's Tau with continuity correction = −0.801, *z* = 4.49, two-tailed *p* < 0.001) indicated the presence of publication bias in this meta-analysis of bariatric surgery effects on circulating ox-LDL. Trim-and-fill analysis revealed that among all included papers in meta-analysis, there could be five missing studies. The “fail-safe N” test showed that 817 missing studies were required to reduce the effect size to a nonsignificant (*p* < 0.001) value (Figure 5).

## 4. Discussion

The results of this meta-analysis revealed a substantial decrease of circulating ox-LDL after bariatric surgery. Of importance, the results of meta-regression did not reveal any significant relationship between the changes in baseline BMI, duration of follow-up or baseline ox-LDL value, and ox-LDL after bariatric surgery. However, there was significant association between the changes in ox-LDL after bariatric surgery and percentage of BMI change.

Some authors have tried to explain the beneficial effect of bariatric surgery on ASCVD by decreasing not only body weight, oxysterols, and ox-LDL but also by decreasing plasminogen activator inhibitor-1 (PAI-1), which is elevated in extremely obese patients and by decreased proliferation of vasa vasorum [35]. Bariatric surgery is a well-documented technique for weight loss can have numerous favorable consequences, such as rise in GLP-1 and its potential function in the metabolism including remission of T2DM [36]. Furthermore, bariatric surgery may be a useful therapy for people who have cardiovascular risk factors [37]. Also, bariatric surgery decreases oxidative stress parameters and glycoproteins and increases antioxidant enzymes paraoxonase-1 and catalase as well, which supports the idea that this procedure decreases oxidation of lipoproteins thus having antiatherogenic effects [34, 35].

Others have tried to explain the beneficial effect of bariatric surgery, besides reducing oxysterols and ox-LDL, by increasing not only HDL-cholesterol but also the number of larger HDL particles which are more atheroprotective and reducing the number of small HDL particles which are less protective [9]. Bariatric surgery, apart from decreasing ox-LDL, also reduces the levels of triglycerides and often decreases the number of LDL particles with a decreased proportion of smaller, more atherogenic LDL particles [38]. Already two decades ago, it has been proposed that small dense LDL particles confer greater risk for atherosclerosis and ASCVD than large, buoyant LDL particles, which might be attributed to the greater oxidative vulnerability of small dense LDL [39].

BMI is an indicator of general obesity and demonstrates significant associations with CVDs and ASCVD risk factor. However, as previously suggested, our data support a direct relationship between the magnitude of improvement in cardiovascular risk factors and the amount of BMI reduction [40]. These consequences may be related to positive correlation between change in serum LDL—as a marker of cardiovascular status—and change in BMI [41]. Furthermore, weight loss-induced LDL reduction might decrease the circulating level of the substrate (i.e., LDL particles) for oxidation, and this could partially account for reduction in the generation of ox-LDL [42]. Also, some think that waist circumference, which has been shown to be associated with elevated ASCVD risk, is a better indicator of abdominal obesity [43]. The others suggest that waist-to-hip ratio is even better for measuring abdominal obesity, and it has been proven that this measure corresponds with ASCVD [44].

The favorable effects of bariatric surgery are most likely to be multifactorial [45]. It should be highlighted that weight reduction is not always required to see improvements in some of with ASCVD risks. Indeed, there is considerable evidence to imply that bariatric surgery may cause changes in oxidative stress, inflammation, and adipokines via nonweight loss pathways [28].

Also, this rather surprising issue that bariatric surgery can improve markers of cardiovascular disorders among patients with severe obesity as long as body fat mass is redistributed or replaced by muscle mass independent of significant weight loss needs further investigation.

This study has some advantages. One of them is that this is the first meta-analysis trying to establish the effect of bariatric surgery on circulating ox-LDL in patients with severe obesity. Further investigations should focus on this premise. This meta-analysis has some limitations as well. The studies which were included had an overall relatively small number of patients. Also, there is heterogeneity in types of operations performed, with known differences in metabolic effects between operations. Also, the methods for measuring ox-LDL concentrations in some studies included in this meta-analysis were different and might have explained heterogeneity in our findings. Besides, the quality of the evidence which was evaluated with GRADE approach was low. Additionally, the time intervals and follow-up compliance are not known in some of the reported studies.

In conclusion, bariatric surgery seems to cause a decrease of circulating ox-LDL which is associated with percentage of BMI change, baseline BMI, duration of follow-up, or baseline ox-LDL value. Future studies may focus on the potential neurohormonal effects that could contribute to this reduction, both dependent and independent of weight loss factors.

## Figures and Tables

**Table 1:** Studies measuring ox-LDL included in the meta-analysis.

Author, year, country	Design of study	Follow-up	Type of surgery	Control (*n*)	Outcome		Patients' characteristic Age Sex	No. of patients
					ox-LDL methods of ox-LDL assessment	BMI change (%)		
Carmona-Maurici et al., 2020 [24] Spain	Prospective observational cohort study	6 months 12 months	Laparoscopic RYGB or SG	**—**	Significant decrease in ox-LDL levels (Mercodia ox-LDL kit)	-32.27	Obese patients with atheromatous plaque 51.8 ± 1.8 years old 18 (F)/14 (M)	32
						-33.91	Obese patients without atheromatous plaque 43.5 ± 1.8 years old 29 (F)/5 (M)	34
Ho et al., 2021 [25]	Prospective, observational study	6 months 12 months	RYGB, SG, or omega loop bypass	Patients seeking weight management [16]	Unchanged (Mercodia ox-LDL kit)	-29.95	Morbid obesity patients 50.1 ± 10 years old -	59
Coimbra et al., 2019 [26]	Observational study	13 months	Laparoscopic adjustable gastric banding (LAGB)	Healthy volunteers [17]	Significant decrease in ox-LDL levels (Mercodia ox-LDL kit)	-11.85	Obese patients 49.03 ± 10.71 years old 18 (F)/2 (M)	20
Gomez-Martin et al., 2018 [27]	Observational study	6 months 12 months	SG	Women matched for age and cardiovascular risk (modified Mediterranean diet) [18]	Significant decrease in ox-LDL levels after 12 months in comparison to baseline and control group (Mercodia ox-LDL kit)	-28.83	Obese women 46 ± 9 years old	20
			Laparoscopic RYGB			-33.82	Obese women 48 ± 8 years old	20
Kelly et al., 2016 [28]	Longitudinal cohort	6 months 12 months	RYGB or vertical SG	**—**	Significant decrease in ox-LDL levels at 12 months (Mercodia ox-LDL kit)	-32.6	Adolescents with severe obesity 16.5 ± 1.6 years old 10 (M)/29 (F)	39
		3 months 12 months	Laparoscopic RYGB	**—**	Unchanged (Mercodia ox-LDL kit)	-35.6	Adolescents with severe obesity 16.5 ± 1.6 years old 3 (M)/10 (F)	13
Müller-Stich et al., 2015 [29]	Prospective cohort	6 months 12 months	RYGB	—	Unchanged (OxiSelect MDA-LDL-quantitation kit)	-25	Patients with BMI more than 35 kg/m^2^ and insulin-dependent T2DM 58.6 ± 6.1 years old 10 (M)/10 (F)	20
Van der Schueren et al., 2015 [30]	Observational study	4 months 7 years	Laparoscopic RYGB	Lean controls [24]	Significant decrease in ox-LDL levels at 7 years (Mercodia ox-LDL kit)	-26.6	Obese patients 40 ± 14 years old 5 (M)/12 (F)	17
Julve et al., 2014 [31]	Observational study	6 months 12 months	RYGB	**—**	Significant decrease in ox-LDL levels (Mercodia ox-LDL kit)	—	Obese patients 20-60 years old 15 (F)/6 (M)	21
Martín-Rodríguez et al., 2014 [32]	Prospective cohort	12 months	Bariatric surgery	Lean Control subjects [30]	Significant decrease in ox-LDL levels (immunodiagnostic system)	-31.37	Obese patients without metabolic syndrome 40 ± 9 years old 19(F)/4(M)	23
						-16.33	Obese patients with metabolic syndrome 42 ± 10 years old 34(F)/5(M)	39
Garrido-Sánchez et al., 2008 [33]	Observational study	7 months	Biliopancreatic diversion, or RYGB	Healthy, nonobese persons [11]	Significant decrease in ox-LDL levels (Mercodia ox-LDL kit)	-33.9	Morbidly obese patients with:	21
							Normal fasting glucose 39.5 ± 11 years old 14(F)/7(M)	
						-30.39	Impaired fasting glucose 44.1 ± 10.6 years old 21(F)/10(M)	31
						-30.74	Type 2 diabetes 44.5 ± 7.4 years old 14(F)/7(M)	21
Uzun et al., 2004 [34]	Observational study	6 months	Open Swedish adjustable gastric band (SAGB)		Significant decrease in ox-LDL levels (Mercodia ox-LDL kit)	-24.24	Morbidly obese patients 35.1 ± 13.1 years old 10(F)/10(M)	20
			Laparoscopic SAGB	—	Significant decrease in ox-LDL levels (Mercodia ox-LDL kit)	-24.27	Morbidly obese patients 34.6 ± 9 years old 11(F)/9(M)	20

**Table 2:** Quality of bias assessment of the included papers in accordance with the Newcastle-Ottawa scale.

Study	Selection				Comparability^†^	Exposure		
	Case definition	Representativeness of the cases	Selection of controls	Definition of controls	Comparability of cases and controls	Ascertainment of exposure	Same method of ascertainment	Nonresponse rate
Ho et al. 2021	—	—	—	—	—	∗	—	—
Carmona-Maurici et al. 2020	—	∗	—	—	—	∗	—	—
Coimbra et al. 2019	—	—	—	∗	—	∗	—	—
Gómez-Martín et al. 2018	—	—	—	—	∗	∗	—	—
Kelly et al. 2016	—	—	—	—	—	∗	—	—
Van der Schueren et al. 2015	—	—	—	∗	∗	∗	—	—
Müller-Stich et al. 2015	—	—	—	—	—	∗	—	—
Martín-Rodríguez et al. 2014	—	—	—	—	—	∗	—	—
Julve et al. 2014	—	—	—	—	—	∗	—	—
Garrido-Sánchez et al. 2008	—	∗	—	∗	—	∗	—	—
Uzun et al. 2004	—	—	—	—	—	∗	—	—

^†^Only for comparability a maximum of two stars can be given.

**Table 3:** Grade assessment.

Summary of findings:						
Effect of bariatric surgery on circulating levels of oxidized low-density lipoproteins in obese patients						
Patient or population: obese patients Setting: - Intervention: bariatric surgery Comparison: -						

Outcome no. of participants (studies)	Relative effect (95% CI)	Anticipated absolute effects (95% CI)			Certainty	What happens
				Difference		

Ox_LDL levels (ox-LDL) assessed with: ELISA/Mercodia/immunodiagnostic/OxiSelect follow-up: range 6 months to 7 years no. of participants: 470 (11 observational studies)	—	The mean ox-LDL levels were 0	—	0 (0 to 0)	⨁⨁◯◯ Low^a,b,c^	
∗The risk in the intervention group (and its 95% confidence interval) is based on the assumed risk in the comparison group and the relative effect of the intervention (and its 95% CI). CI: confidence interval						

GRADE working group grades of evidence High certainty: we are very confident that the true effect lies close to that of the estimate of the effect. Moderate certainty: we are moderately confident in the effect estimate: the true effect is likely to be close to the estimate of the effect, but there is a possibility that it is substantially different. Low certainty: our confidence in the effect estimate is limited: the true effect may be substantially different from the estimate of the effect. Very low certainty: we have very little confidence in the effect estimate: the true effect is likely to be substantially different from the estimate of effect.						

## Data Availability

There is not any raw data associated with this review article.

## Additional Points

*Key Points*. (i) Obesity is related to increased circulating oxidated LDL (ox-LDL) and atherosclerotic risks. (ii) Bariatric surgery may lower the risk of cardiovascular mortality. (iii) Bariatric surgery in severely obese causes a decrease of circulating ox-LDL.
